# Nephrogenic diabetes insipidus in a geriatric patient affected by SARS-CoV-2: complexity of a diagnosis, complexity of a virus

**DOI:** 10.1099/acmi.0.000598.v4

**Published:** 2024-01-31

**Authors:** Maria Ada Corich, Michele Niero, Lucia Corich, Elisabetta Ferretti, Paolo De Colle, Stella Bernardi, Giuliano Ceschia, Gianfranco Sanson, Michela Zanetti

**Affiliations:** ^1^​ Geriatric Unit, Azienda Sanitaria Universitaria Giuliano Isontina, Trieste, Italy; ^2^​ Postgraduate School in Geriatrics, Department of Medical, Surgical and Health Sciences, University of Trieste, Trieste, Italy; ^3^​ Microbiology and Virology Laboratory, Azienda Sanitaria Friuli Occidentale, Pordenone, Italy; ^4^​ SS Endocrinologia, UCO Medicina Clinica, Cattinara University Hospital, Trieste, Italy; ^5^​ Department of Medical, Surgical and Health Sciences, University of Trieste, Trieste, Italy

**Keywords:** severe acute respiratory syndrome coronavirus 2, nephrogenic diabetes insipidus, inflammation, angiotensin 2 converting enzyme, transmembrane serine protease type 2, polyuria, urinary osmolarity

## Abstract

**Background.:**

Coronavirus disease 2019 (COVID-19) has an important impact on the kidney through direct and indirect damage mechanisms. Most previous studies have highlighted lesions caused by this virus in the early segments of the nephron. However, due to the antigenic characteristics of the virus, with almost ubiquitous receptors, and the molecular release it triggers, the distal segments of the nephron could also be affected.

**Methods.:**

A 71 year-old-man with respiratory failure while suffering from severe acute respiratory syndrome coronavirus 2 (SARS-CoV-2) pneumonia presented with typical symptoms of diabetes insipidus after ~20 days of hospitalization. The water deprivation test led to the diagnosis of nephrogenic diabetes insipidus. The aetiological study was complex, in particular because of the patient’s previous lithium therapy.

**Results.:**

The sequence of pathognomonic events typical of diabetes insipidus associated with anamnestic, clinical and laboratory evidence strongly supported the diagnosis of nephrogenic diabetes insipidus due to SARS-CoV-2 rather than other aetiologies.

**Conclusions.:**

The collecting duct could represent a target for SARS-CoV-2 infection, directly or indirectly, as a result of lesions of upstream portions of the nephron, which would cascade into the distal segment. Other molecules, besides angiotensin 2 converting enzyme, might be involved in facilitating the viral aggression. The complexity of the geriatric patient shows the importance of a comprehensive approach that integrates careful monitoring of clinical signs and symptoms and laboratory and instrumental tests. This is especially important in the context of SARS-CoV-2 infection and in the management of its unexpected complications.

## Data Summary

No data were required for the work to be reproduced.

## Introduction

Since the end of 2019, severe acute respiratory syndrome coronavirus 2 (SARS-CoV-2) has been responsible for a global health emergency, with millions of infections worldwide. Globally, since the beginning of the pandemic, there have been 772166517 confirmed cases with 6981263 deaths (update: 22 November 2023) [1], of which 26577519 (192279 deaths) were in Italy (update: 22 November 2023) [2].

Although the respiratory system is the main target of the virus, several organs and apparatuses can be involved, resulting in multiorgan dysfunction [3–5], mainly due to the syndrome from cytokine release [6, 7]. Several cases of SARS-CoV-2-associated acute renal injury were reported [8, 9]. They were linked to direct and indirect mechanisms, including binding of the virus to the angiotensin 2 converting enzyme (ACE2) or other receptors, such as transmembrane serine protease type 2 (TMPRSS2), cathepsin L (CTLS) and cluster of differentiation 147 (CD147), as well as imbalance in the renin–angiotensin–aldosterone system (RAAS), cytokine activation and thrombotic events [9].

So far, evidence suggests that coronavirus disease 2019 (COVID-19)-related nephropathy predominantly involves the proximal nephron tract. Involvement of the distal segment resulting in nephrogenic diabetes insipidus (NDI) seems unusual, since it has been reported exclusively in intensive care units, with the concomitant use of sevoflurane [10]. However, this finding suggests the expansion of molecular analysis beyond the ACE2 – expressed mainly at the glomerular and tubule proximal level [9, 11] – considering other molecules present in several portions of renal tubulus potentially able to favour cell aggression by SARS-CoV-2 [12].

Diabetes insipidus is characterized by the excretion of large urinary volumes (polyuria, described as urinary volume >2 l m^−^² 24 h^−1^ [13]) with low osmolarity and low specific gravity, together with an intense thirst.

It may be caused either by a deficiency of antidiuretic hormone (ADH) secretion by the hypothalamus and posterior pituitary gland (central diabetes insipidus) or by a renal resistance to the activity of ADH (nephrogenic diabetes insipidus).

Another cause of polyuria with low osmolarity and low specific gravity is excessive intake of liquids (primary polydipsia).

The diagnosis is confirmed by means of the water deprivation test.

We present the case of a 71-year-old man who developed NDI associated with SARS-CoV-2-related pneumonia during hospitalization for respiratory failure (May 2021). To our knowledge this complication has not been previously documented in the literature.

## Methods

### Premise

The patient’s medical history included chronic kidney failure class KDIGO 3 on the nephroangiosclerotic basis, arterial hypertension, peripheral vasculopathy of the lower limbs with occlusion of the left common iliac artery, chronic psychosis and chronic obstructive pulmonary disease in an active smoker. Two recent hospitalizations due to urinary infection (October 2020) and community-acquired pneumonia (January 2019) were reported, with secondary transient worsening of renal function in both cases.

There was no evidence of previous hospitalizations due to dehydration, except as a result of infective episodes. No recent head trauma was documented.

From the interview with the caregivers, no previous symptoms ascribable to diabetes insipidus (e.g. polydipsia) were reported; conversely, low spontaneous water intake was described. The previously performed blood tests did not show frank hypernatraemia.

Medications included acetylsalicylic acid 100 mg, silodosine 8 mg and risperidone 1 mg; lithium therapy had been discontinued 2 years before, because of overdose with acute kidney failure.

### In the emergency department

The patient was referred to the Emergency Department because of persistent fever and dysuria despite antibiotic treatment for a recent urinary tract infection. The RT-PCR analysis performed on nasopharyngeal swab showed positivity for SARS-CoV-2. The patient received intravenous hydration and was transferred to a nursing home for clinical monitoring and treatment. Blood and urine test results and volume of fluids at admission and during hospital stay are shown in Table 1.

**Table 1.** Laboratory data

	In the emergency room	During hospitalization (19th day)	At transfer to non-COVID medicine (44th day)
Urea (mg dl^−1^)	81	35	60
Creatinine (mg dl^−1^)	2.5	1.55	2.07
Sodium (mEq l^−1^)	147	151	143
Potassium (mEq l^−1^)	4.12	4.03	5.2
Plasma osmolarity (mOsm kg^−1^)	–	305	292
Urine osmolarity (mOsm kg^−1^)	–	231	172
Urine specific gravity (g l^−1^)	–	1006	1008
Hydration (ml)	–	1920	2500
Diuresis (ml)	–	2300	2800

### In the nursing home

Twenty hours later, at nursing home admission, he presented with lethargy, deteriorated cognitive status and dysphagia, normal oxygen saturation in ambient air and clear lungs on auscultation. The following day steroids (methylprednisolone 40 mg i.v.), prophylactic enoxaparine and low-flow oxygen therapy were initiated because of altered breath sounds and initial desaturation. Chest X-ray showed an uneven parenchymal thickening at right lung bottom and many radiopaque small spots bilaterally at midbase fields. Antibiotic therapy with amoxicillin/clavulanic acid and clarithromycin was initiated.

Meanwhile, renal function deteriorated despite infusion of 5% dextrose solution up to 100 ml h^−1^ after nephrology consultation, while 24 h diuresis was ~1500 ml.

Given the need for closer monitoring of the hydro-electrolytic balance, the patient was transferred to the Geriatric Department after 8 days in the nursing home.

### In the geriatric unit

At arrival, he was alert and able to execute simple orders. His blood pressure was 100/80 mmHg, heart rate 70 b.p.m., rhythmic and SpO_2_ 96% with 4 l of oxygen. A bladder catheter was placed, revealing clear urine. Skin and mucous membranes were dry. The vesicular murmur was broadly reduced. The patient presented with seborrheic skin on the face, amimic facies, reduced muscular trophism and plastic hypertone to the four limbs, with trochlea particularly noticeable on the wrists, tremor resting on the left hand; lower and upper limbs Mingazzini test was negative. The electrocardiogram showed sinus rhythm, with QRS axis of +30°.

The blood tests showed: creatinine 2.89 mg dl^−1^, sodium 156 mEq l^−1^, potassium 4.32 mEq l^−1^, albumin 2.88 g dl^−1^, PCR 35.9 mg dl^−1^, d-dimer 1474 ng mLFEU^−1^, haemoglobin 11 g dl^−1^ and procalcitonin 0.29 ng ml^−1^.

Steroid therapy and enoxaparin were continued. The antibiotic was switched to piperacillin/tazobactam, adjusting the dose based on renal function, with a rapid clinical improvement of pneumonia. There were no clinical suspicions for either deep vein thrombosis or pulmonary embolism. Gas exchange was not suggestive of pulmonary embolism.

After stopping risperidone, the clinical picture of Parkinsonism improved, in particular the plastic hypertone and dysphagia, with recovery of swallowing ability. Further, the patient was progressively more reactive and showed greater ability to interact. The patient underwent physiotherapy, soon recovering autonomy in postural steps and walker-assisted gait.

The patient received hydration initially with 5% dextrose solution and then with Ringer’s acetate and finally with isolyte, adjusting the infusion rate on the basis of the calculated water deficit [14], with progressive improvement of renal function, with daily diuresis of about 2000 ml, serum creatinine 1.5 mg dl^−1^ and glomerular filtration rate (GFR) 44 ml min^−1^ according to Berlin Initiative Study-1 (BIS1).

## Results

**Fig. 1.**
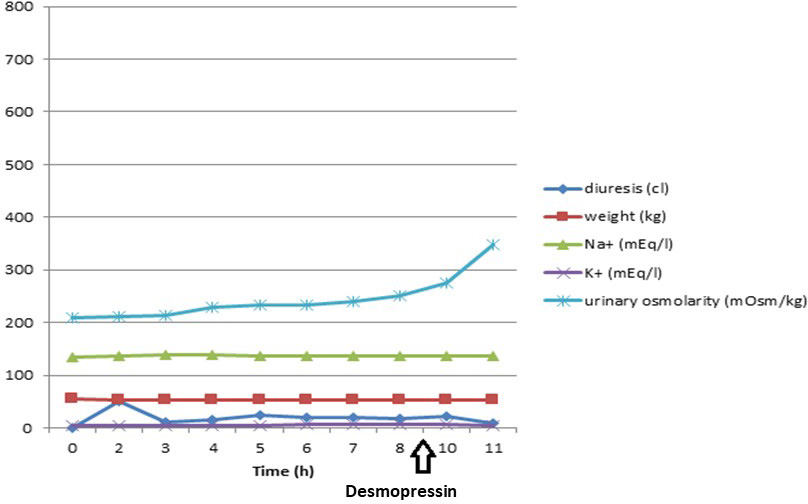
Time course of significant clinical and laboratory parameters during water deprivation test.

Surprisingly, the urinary volume, previously ~2000 ml/day, 18 days after admission increased to ~5000 ml/day, with a peak of 7300 ml in 24 h on the 25th day, in the absence of diuretic therapy. Urine presented low specific gravity and hyposmolarity, while increased serum osmolarity and persistent serum hypernatraemia were observed. In particular, on the 23rd day urine osmolarity and specific gravity were 231 mOsm kg^−1^ and 1006 g l^−1^, respectively, while plasma osmolarity was 305 mOsm kg^−1^ and natremia 149 mEq l^−1^. A provisional diagnosis of diabetes insipidus was formulated. On the 26th day a water deprivation test was performed, which confirmed the diagnosis (Fig. 1 and Table 2). The lack of a significant increase in urine osmolarity (excluding the diagnosis of polydipsia) and the modest increase of urine osmolarity 1 and 2 h after i.v. administration of desmopressin 2 µg led us to conclude that this was the nephrogenic form. Therefore, water supplementation therapy was started with benefit. This diagnosis has been also supported by the fact that a few months later the patient was hospitalized again for hypovolemic shock from serious dehydration with a natremia equal to 181 mEq l^−1^.

**Table 2.** Water deprivation test for differential diagnosis among polydipsia, central diabetes insipidus and nephrogenic diabetes insipidus

Time (h)	Weight (kg)	Diuresis (ml)	Plasma osmolarity (mOsm kg^−1^)	Urinary osmolarity (mOsm kg^−1^)	Na + (mEq l^−1^)	K + (mEq l^−1^)
0	54.5	0	284	210	135	5.06
2	53.9	500	285	211	136	4.8
3	53.65	120	287	214	139	5.2
4	53.45	150	285	229	138	5.45
5	53.4	250	286	234	136	5.41
6	53.05	200	286	233	137	5.68
7	52.8	200	289	241	136	5.87
8	52.35	170	287	250	136	5.85
9	**Administration of 2 µg of desmopressin**					
10	–	210	287	275	136	5.69
11	51.95	80	287	347	137	5.3

In light of the result of the water deprivation test, the endocrinology was consulted. It confirmed the diagnosis of nephrogenic diabetes insipidus and recommended free access to water, calculating a water requirement of approximately 2.5 l per day and the introduction of thiazide (hydrochlorthiazide 25 mg 1 tablet) if hypernatraemia persisted.

## Discussion

The commonly reported complications associated with COVID-19 include myocarditis, pericarditis, restrictive lung disease, thromboembolic events, total or partial loss of taste and smell, anxiety, depression, post-traumatic disorder, chronic fatigue and myalgia [15], as well as central diabetes insipidus [16]. However, at present, many complications of COVID-19 are still unknown.

Here we describe for the first time the case of a patient who presented with NDI 2 weeks from the diagnosis of COVID-19-related pneumonia. Although SARS-CoV-2 infection could not be identified with certainty as the only cause of NDI, there are many reasons to suspect its active role in the aetiology of this condition.

Many studies have highlighted the involvement of the kidney in this disease, resulting in SARS-CoV-2-related nephropathy. Different degrees of clinical manifestations were described, from alterations in urinary sediment (haematuria, proteinuria, hyperkaliuria), to severe forms of AKI requiring dialysis treatment [8, 9, 14]. In most cases, low-grade proteinuria – probably due to a tubular damage – was documented, although high proteinuria or albuminuria were uncommon, suggesting a glomerular problem [9].

From a histopathological point of view, a post-mortem study on 26 patients who died from COVID-19 described the presence of acute tubular damage in optical microscopy. Another study employing electron microscopy found the presence of viral particles both inside the renal tubule cells and in the glomerular podocytes of a single patient [11]. In addition, another autopsy study on six patients revealed the presence of viral particles in all examined kidney portions [12].

With regard to molecular analysis, a large expression of ACE2, TMPRSS2 and CTSL, considered entry doors for SARS-CoV-2 in the cells, has been documented in different portions of renal tubules [17]. The presence of the transmembrane receptor CD147, which has recently been identified as a possible gateway for SARS-CoV-2 to enter the cell through a specific spike protein, has also been found ubiquitously in the kidney [9, 18].

More precisely, ACE2 seems to be more expressed in the glomerulus and proximal tubule [9, 17], although it is also present in the collecting duct [19]; TMPRSS2 seems to be very represented in the distal portions of the renal tubule [8] and CD147 is more expressed in the proximal renal tubule, although detected in all portions [9].

A recent consensus has classified the direct and indirect possible mechanisms of SARS-CoV-2-induced renal damage [20]. They highlight a multifactorial aetiology of this finding.

**Fig. 2.**
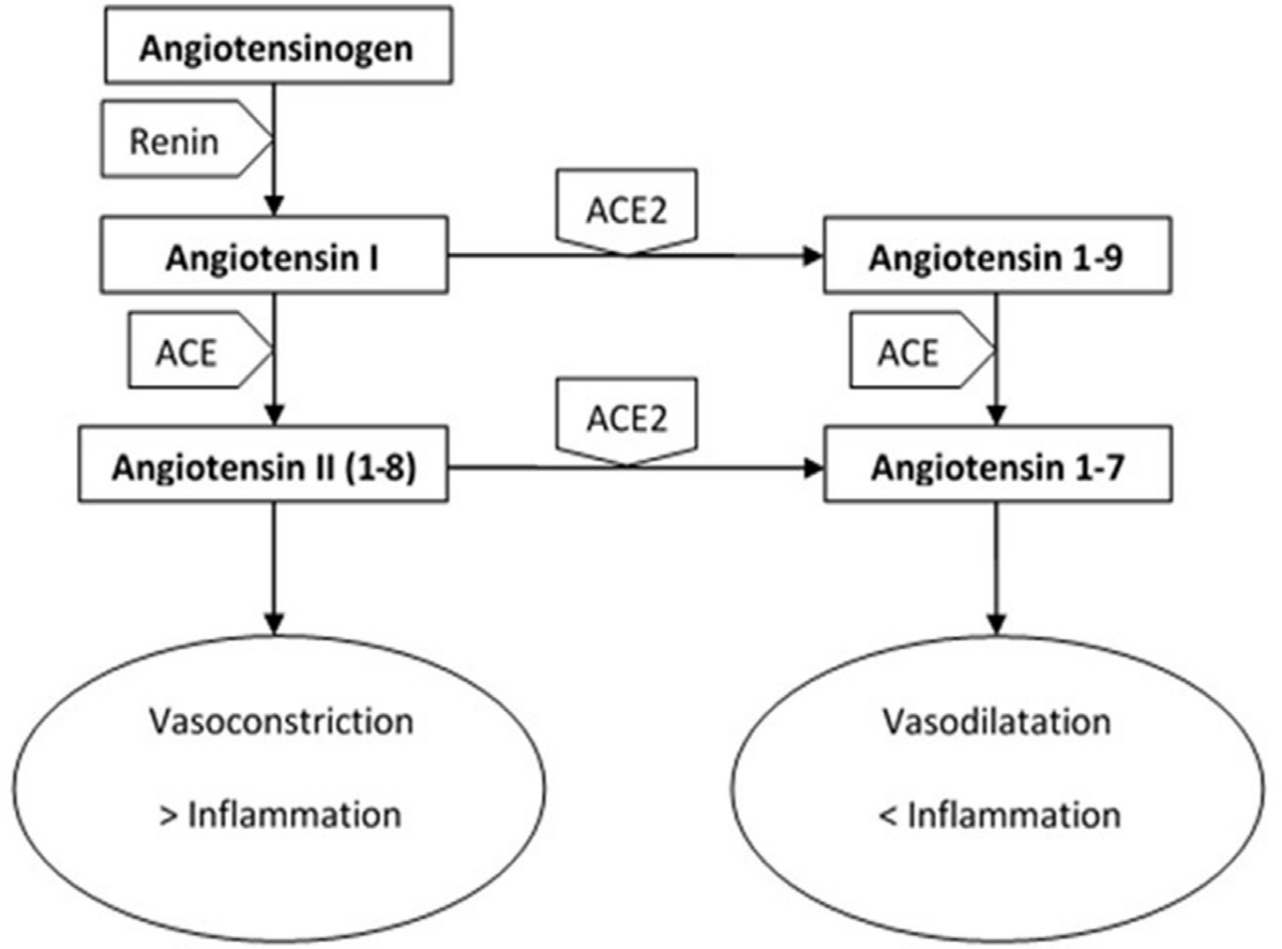
Role of ACE2 in the RAAS.

The direct mechanisms include both a cellular aggression due to the entry of the virus through the receptors described above and a reduction of ACE2 expression on cell membranes induced by the virus (through binding to the receptor itself) with consequent accumulation of angiotensin 2 and hyperactivation of RAAS; the latter phenomenon leads to a worsening of the inflammatory state, a tendency to fibrosis and vasoconstriction, a reduction in natriuresis and an increase in sodium reabsorption [9, 21] (Fig. 2).

A third direct mechanism is represented by overexpression and activation of many inflammatory cytokines, which can cause important dysfunctions in renal cells at the endothelial and tubular level; e.g. TNF-α, through its receptors in the tubule, can induce cellular apoptosis.

Finally, a further direct mechanism is the induction of dysfunctions in renal microcirculation, due to the prothrombotic state linked to the disease, as demonstrated by the finding of fibrin deposits in the glomerular loops. The hypothesis of the involvement of renal microcirculation in COVID-19 is also supported by the finding of diffuse erythrocyte aggregation and obstruction in the lumen of glomerular and peritubular capillaries without platelets, red blood cell fragments, fibrin thrombi or fibrinoid necrosis.

Moreover, angiotensin II accumulation may promote an imbalanced RAAS activation, promoting vasoconstriction [22].

The indirect mechanisms of kidney damage are those related to the systemic effects of the virus or to the involvement of the kidney in virus-related damage to other organs. Some examples are fluid loss, due to hyperpyrexia or the gastrointestinal manifestations of COVID-19, which can contribute to AKI; the use of nephrotoxic drugs for COVID-19 treatment, such as some antibiotics; or the release into circulation of DAMPs by other damaged organs, such as the lung [20, 23].

It is therefore clear that, due to characteristics previously described in our patient, there are several pathogenetic mechanisms that may have led to the development of NDI.

In addition, it should be noted that our patient had previously undergone chronic lithium therapy, although this was stopped ~2 years before due to the onset of AKI. Consequently, while discussing a possible SARS-CoV-2-linked aetiology of NDI, the role played by lithium should also be considered. Notably, chronic use of lithium is one of the most frequent causes of NDI onset. In this case, drug-related damage can be mediated by various molecular mechanisms, which act in particular in the internal medullary area of the collecting duct [24], mainly by blocking at various levels the vasopressin receptor signal transduction cascade or through the reduction of its expression, leading to a reduced expression of AQP2 in the main cells of the collecting duct [24].

As indicated by several studies, NDI is usually a concomitant event with lithium intake, with few cases showing onset of NDI even after several years of drug suspension, mainly in the post-surgical setting [25] or in patients with limited access to hydration [26].

This appears to be due to a slow recovery of AQP2 gene expression after lithium suspension, a loss of the renal medullary osmotic gradient, or a lithium-induced interstitial nephritis resulting in persistent renal failure [25]. The disease, which had remained silent until that moment, would be revealed by a limitation on free access to water.

Our patient, unlike those mentioned above [25, 26], had free access to water, both before and during hospitalization. Furthermore, he had been widely hydrated during the hyperpyrexia phase.

It is worth noting that neither the patient nor his family members had reported the presence of polydipsia or hypernatremia before hospital admission and hypernatremia was not documented by previous laboratory data. In addition, during previous hospitalizations, serum sodium had never been significantly elevated, even during dehydration induced by pyrexia and inflammation.

Finally, polyuria was not evident during hospitalization in the intermediate care facility and in the first phase of hospitalization in our unit, as demonstrated by normal diuresis and water intake monitoring. We can therefore conclude that the NDI developed by our patient may be due to SARS-CoV-2 viral infection, rather than to the previous use of lithium.

The peculiar complexity of geriatric medicine, which requires the unravelling of aspects that are often tangled up with the medical and pharmacological history and the actual clinical patient situation, was evident in this case. It is also clear how continuous clinical observation, parametric and laboratory monitoring, and involvement of family members are mandatory in order to guide us towards a correct diagnosis.

## Conclusions

The presented case, along with the review of the cited literature, underline the multifactorial aetiology of kidney damage induced by Sars-CoV-2.

This work analyses both the role of previous lithium therapy and the direct damage induced by Sars-CoV-2 on the lesion to the collecting tubule. It also discusses the reasons that support the diagnosis of nephrogenic diabetes insipidus caused by Sars-CoV-2 rather than lithium in our patient.

In particular, SARS-CoV-2 infection may have revealed the damage to the collecting duct, previously predisposed by the chronic use of lithium.

Physiologically, water absorption in the renal collecting duct is regulated by arginine vasopressin, through its V2 receptor and cyclic AMP signalling, resulting in increased expression of aquaporin-2 and its apical membrane targeting in the renal collecting duct cells. The arginine vasopressin–V2 receptor–aquaporin 2 pathway is disrupted in acquired NDI, resulting in the development of resistance of the renal collecting duct to arginine vasopressin [27].

Lithium inhibits vasopressin-stimulated cyclic adenosine monophosphate, reducing urinary concentrating ability [28].

The pathogenesis of central diabetes insipidus is the deficit of arginine vasopressin and not cellular resistance to it.

Instead, in favour of a purely viral damage, we emphasize that the chronology of the manifestations of NDI seems to correlate more with SARS-CoV-2 infection than with the use of lithium. Moreover previous infections did not result in a characteristic pattern of NDI, unlike what occurred after SARS-CoV-2.

Finally we speculate that the collecting duct could represent a receptor target in SARS-CoV-2-related infection, being particularly vulnerable to the pathogenetic mechanism triggered by COVID-19; other molecules, in addition to ACE2, whose role as a receptor for the virus is well known, may represent gateways and be implicated in the pathogenesis of kidney damage, even in the distal portions of the nephron. This would be supported by their greater presence in these areas with consequent possible direct damage. Lastly, considering that renal damage from SARS-CoV-2 affects different parts of nephron, we could hypothesize that the involvement of the collecting duct, the last part of the functional unit, may also be a consequence of a damage cascade in the previous sectors.

Further molecular analyses at the receptor level of the collecting duct should be performed in relation to the possible sites of action of the virus and autopsy observations could help to understand whether Sars-CoV-2 damage to the proximal parts of the nephron can lead to alterations in the distal parts of the nephron.
